# Atlanta Medical College

**Published:** 1879-03

**Authors:** 


### Editorial.

---

### ATLANTA MEDICAL COLLEGE.

The commencement exercises of this institution took place on Tuesday night, the 4th of March, before a large and fashionable audience.

After prayer by Rev. Mr. Gwinn, of the First Baptist Church, thirty-five who had passed a satisfactory examination before the faculty, were presented and had the degree of Doctor in Medicine conferred upon them by Col. William L. Mitchell. Three physicians in good standing were admitted *ad eundem gradum* in the college. Their names are as follows:

W. G. Adair, H. D. Allen, F. Beals, J. S. Bennett, B. F. Braselton, F. J. Brooks, J. F. Brooks, W. J. Chapman, E. S. Davis, J. A. Gray, R. R. Harden, J. N. Hogg, Frank Holland, W. W. James, W. B. Leake, T. A. McLarty, J. P. McWilliams, J. T. Mandeville, J. J. Morgan, F. M. Newman, A. G. North, L. H. Peebles, M. V. Pool, J. A. Putnam, A. L. Rayle, J. W. Riley, J. L. Selman, W. L. Sikes, J. T. Slaughter, J. M. Spinks, H. B. Stewart, C. T. Stovall, R. H. Taylor, C. W. Vanvalkenburgh, J. L. Walker.

*Ad eundem*—Javan Bryant, M.D., J. W. Maydon, M D., Maurice Salm, M.D.

Col. Boynton, of Griffin, was then introduced, and delivered the valedictory address to the graduating class, as published in the present number of this JOURNAL. This was responded to on the part of the graduates by Dr. W. W. James, one of their number. This will also be found in full on our pages.

The following prizes were awarded by the faculty and presented by Rev. Mr. Gwinn:

By the faculty for the highest grade of proficiency as exhibited in the general examination, a case of surgical instruments, which was awarded to Dr. J. A. Gray. Honorable mention was also made of Drs. R. H. Taylor, C. T. Stovall and L. H. Peebles.

Prof. J. G. Westmoreland offered a medical case for the best thesis on the action of remedies, which was awarded to Dr. J. F. Brooks.

Prof. W. F. Westmoreland offered a transfusion apparatus for the best report of the surgical clinic, which was awarded to Dr. H. D. Allen.

Prof. V. H. Taliaferro offered a set of forceps for the best examination in his department, which was awarded to Dr. R. H. Taylor.

Prof. Calhoun offered an ophthalmoscope for the best report of the eye and ear clinic, which was awarded to Dr. C. T. Stovall.

Prof. Johnson offered a case of instruments for the best thesis on the operative treatment in his department, which was awarded to Dr. J. A. Gray.

Prof. Logan offered a gold medal for the best thesis on the relation of chemistry to the science of medicine, which was awarded to Dr. H. B. Stewart.

---

We publish the following from the *Bulletin of the Public Health*, issued by John M. Woodworth, the Surgeon-General U. S. Marine Hospital Service, under the National Quarantine Act of 1878, for the week ending February 22d, 1879:

OFFICE SURGEON-GENERAL M. H. S,
WASHINGTON, Feb. 26, 1879.

*Boston.*—Week ended Feb. 22. Deaths from all causes, 139, an annual ratio of 20 per 1,000 of the population; 14

cases of scarlet fever, 6 deaths; 20 cases of diphtheria, 7 deaths. Bronchitis caused 7 deaths, pneumonia 10, phthisis 31.

*Providence.*—Week ended Feb. 22. Total deaths 29; annual ratio 15; 1 death from diphtheria, 2 from scarlet fever.

*New York.*—Week ended Feb. 22. Total deaths 551; annual ratio 26.3; 3 deaths from enteric fever, 50 from scarlet fever, 15 from diphtheria, 18 from croup, 91 from pneumonia and bronchitis, 92 from phthisis.

*Brooklyn.*—Week ended Feb. 22. Total deaths 210; ratio 19.34; 83 cases of scarlet fever, 9 deaths; 40 cases of diphtheria, 11 deaths; 3 deaths from enteric fever, 31 from acute pulmonary diseases, 26 from phthisis.

*Buffalo.*—Week ended Feb. 22. Total deaths 36; annual ratio, 13; 4 deaths from scarlet fever, 5 from diphtheria, 2 from enteric fever, 16 from phthisis.

*Philadelphia.*—Week ended Feb. 22. Total deaths 353; annual ratio 21.2. Enteric fever caused 10 deaths, scarlet fever 7, diphtheria 15, whooping cough 4, acute pulmonary affections 55, phthisis 55. "Pulmonary affections prevalent, diphtheria increasing."

*Pittsburgh.*—Week ended Feb. 22. Total deaths 45; annual ratio 16; 1 death from enteric fever, 1 from scarlet fever, 6 from diphtheria.

*Baltimore.*—Week ended Feb. 22. Total deaths 131; annual ratio 18.66. Enteric fever caused 3 deaths, scarlet fever 3, diphtheria 3, croup 6, acute pulmonary diseases 22, phthisis 29.

*District of Columbia.*—Week ended Feb. 22. Total deaths 75; annual ratio 24.3. Enteric fever caused 2 deaths, scarlet fever 6, diphtheria 1, acute pulmonary diseases 13, phthisis 13.

*Hudson County, N. J.* (including Jersey City and Hoboken).—Week ended Feb. 22. Total deaths, 71; annual ratio 19.6. Scarlet fever caused 6 deaths, diphtheria 4, acute lung affections 13, phthisis 8.

*Richmond.*—Week ended Feb. 22. Total deaths 29; ratio 18.20; 6 deaths from scarlet fever.

*Cincinnati.*—Week ended Feb. 22. Total deaths 101; annual ratio 19; 13 deaths from scarlet fever, 1 from diphtheria.

*St. Louis.*—Week ended Feb. 22. Total deaths 111; annual ratio 21.4. Enteric fever caused 2 deaths, diphtheria 1.

*San Francisco.*—Week ended Feb. 14. Total deaths 85; annual ratio 14.5. Enteric fever caused 2 deaths, diphtheria 3, pneumonia 16, phthisis 8.

*Mobile.*—Week ended Feb. 22. Total deaths 21; annual ratio 27. Acute lung diseases caused 4 deaths.

*New Orleans.*—Two weeks ended Feb. 23. Total deaths 187; annual ratio 23.4. Diphtheria caused 3 deaths, acute lung diseases 29, phthisis 39.

*Island of Bermuda.*—In a population of 15,300, during the six weeks ending Feb. 18, there were 15 deaths, over 50 per cent. being of persons over 80 years of age.

*Havana.*—Week ended Feb. 22. Yellow fever caused 1 death, small-pox 13.

Small-pox is very prevalent in Cuba, Brazil, Dublin, London, St. Petersburg, and the ports of India, and less so at Buda Pesth, Vienna, Paris and Barcelona.

